# Generalized pairwise comparisons of prioritized outcomes are a powerful and patient-centric analysis of multi-domain scores

**DOI:** 10.1186/s13023-023-02943-8

**Published:** 2023-10-12

**Authors:** Vaiva Deltuvaite-Thomas, Mickaël De Backer, Samantha Parker, Marie Deneux, Lynda E. Polgreen, Cara O’Neill, Samuel Salvaggio, Marc Buyse

**Affiliations:** 1 International Drug Development Institute, Avenue Provinciale 30, 1340 Louvain-la-Neuve, Belgium; 2 Interuniversity Institute for Biostatistics and Statistical Bioinformatics (I-BioStat), Hasselt University, Diepenbeek, Belgium; 3 Institut de Statistique, Biostatistique et Sciences Actuarielles, Université Catholique de Louvain, Louvain-la-Neuve, Belgium; 4 Lysogene, Neuilly-sur-Seine, France; 5 Lundquist Institute at Harbor-UCLA Medical Center, Torrance, CA USA; 6 Cure Sanfilippo Foundation, Columbia, SC USA; 7 Present Address: Innoskel, Biot, France

**Keywords:** Generalized pairwise comparisons, Prioritized outcomes, O’Brien test, Net benefit, Multi-domain analysis, Sanfilippo syndrome, Mucopolysaccharidosis type IIIA

## Abstract

**Background:**

Generalized pairwise comparisons (GPC) can be used to assess the net benefit of new treatments for rare diseases. We show the potential of GPC through simulations based on data from a natural history study in mucopolysaccharidosis type IIIA (MPS IIIA).

**Methods:**

Using data from a historical series of untreated children with MPS IIIA aged 2 to 9 years at the time of enrolment and followed for 2 years, we performed simulations to assess the operating characteristics of GPC to detect potential (simulated) treatment effects on a multi-domain symptom assessment. Two approaches were used for GPC: one in which the various domains were prioritized, the other with all domains weighted equally. The net benefit was used as a measure of treatment effect. We used increasing thresholds of clinical relevance to reflect the magnitude of the desired treatment effects, relative to the standard deviation of the measurements in each domain.

**Results:**

GPC were shown to have adequate statistical power (80% or more), even with small sample sizes, to detect treatment effects considered to be clinically worthwhile on a symptom assessment covering five domains (expressive language, daily living skills, and gross-motor, sleep and pain). The prioritized approach generally led to higher power as compared with the non-prioritized approach.

**Conclusions:**

GPC of prioritized outcomes is a statistically powerful as well as a patient-centric approach for the analysis of multi-domain scores in MPS IIIA and could be applied to other heterogeneous rare diseases.

**Supplementary Information:**

The online version contains supplementary material available at 10.1186/s13023-023-02943-8.

## Background

Mucopolysaccharidosis type IIIA (MPS IIIA), or Sanfilippo A syndrome, is a rare monogenic lysosomal disease that causes intracellular accumulation of toxic levels of partially degraded oligosaccharides [20]. MPS IIIA typically causes severe neurodegenerative symptoms that lead to a rapidly deteriorating quality of life for the patient [21]. The estimated prevalence of MPS IIIA falls between 1:250,000 and 1:50,000 depending on the population studied [10]. The majority of patients have the rapidly progressing or severe classical form of MPS IIIA, and typically symptoms are first recognized between 1 and 3 years of age with slowed cognitive development. Most children with MPS IIIA never reach a cognitive age greater than 3 years. They go on to experience severe behavioral problems, loss of speech, and progressive intellectual decline. Later in the teenage years, children lose most intellectual and communicative abilities, experience a decline of all motor functions, which culminates in complete loss of locomotion, dysphagia, and pyramidal-tract lesions [2, 18, 22].

As it is a heterogenous disease profoundly affecting various organs and disrupting multiple functions with its multitude of symptoms, MPS IIIA is a pertinent example highlighting the significant challenges in identifying a single clinically relevant endpoint for trials. Specifically, there may be a range of differential effects of therapy on the varying disease manifestations of MPS IIIA, and these effects may also vary between individuals. Thus, capturing comprehensive change in disease status cannot be adequately measured through a single outcome or endpoint. Three prospective longitudinal natural-history studies have published neurocognitive outcomes in patients with MPS IIIA [2, 18, 20, 24]. Such outcomes are typically assessed using developmental scales, like the Bayley Scales of Infant Development (BSID-III), within which three domains are of focus: cognitive, language, and motor development [1]. The scores across these domains have historically been summarized in the Development Quotient (DQ), which serves as a numerical estimate of a child’s neurocognitive development. An overall general DQ is obtained by dividing the subject’s developmental age (DA) by chronological age (CA), and multiplying by 100 (DQ = DA/CA × 100) [6]. In subjects with MPS IIIA, to accurately capture the impact of disease and treatment, it is important to supplement standardized assessments of neurocognitive development with assessments from other domains, including behavioral capabilities, sleep–wake habits, pain, eating, as well as the quality of life of the subjects and their families [24]. Furthermore, these domains are reported by parents of children with MPS IIIA as the most important to treat according to previous qualitative research using parent interviews [11, 15].

A recent FDA Guidance clearly recommends to “assess multiple, distinct clinical endpoints in trials to provide a global characterization of treatment effects on disease manifestations”[8]. Given that quantitative assessments of benefits (and potential risks) of new treatments are typically estimated using univariate methods, estimation and testing of each outcome individually requires multiplicity correction, generally resulting in a loss of power. Furthermore, since the correlations between the individual outcomes are ignored, it is difficult to gauge the overall effect of a treatment on all the domains of interest simultaneously. As an alternative, to enable assessment of multidimensional domain scores, composite measures such as DQ have historically been used. These scores can capture clinically meaningful changes across multiple domains within individuals and, as such, have better statistical power and clinical relevance than any univariate outcome measure in most cases [19]. The utilization of a single composite score is advantageous for statistical analysis as well through the use of standard statistical tests, facilitating predefined decision-making processes. For instance, a new treatment may be considered eligible for regulatory approval if a minimum difference considered to be clinically important is seen in treated patients as compared with control patients. There are, however, some limitations to these composite outcome analyses: they may lose granularity and lack power to detect the most meaningful outcome among the contained domains, or identify specific outcomes within the domain driving the analysis in opposite directions [4]. More importantly, these composite measures are unlikely to capture all neurological disease manifestations that are important to patients and their families [12, 23].

In this paper, we explore a new method for the analysis of multi-domain scores in comparative trials, using generalized pairwise comparisons (GPC) of all subjects receiving the new intervention *vs.* all subjects receiving the standard of care [3]. Since no randomized clinical trials of new treatments have been conducted in patients with MPS IIIA, we perform simulations based on a natural history cohort of 23 untreated subjects as described in the Methods section. We fit a model to these data, and use this model to generate two sets of hypothetical observations, one set having the same distribution as the natural history cohort, and the other set having shifted distributions to reflect treatment effects that experts would consider plausible and clinically worthwhile. We show the flexibility of GPC to perform analyses that include an arbitrary number of domains (and subdomains if desired), similarly varying thresholds of clinical relevance for each domain, and various types of aggregations of the domains (prioritized with potentially various orders of priorities, and non-prioritized). The choice of the preferred analysis may be highly context-dependent, but the approach remains identical from a mathematical as well as from an interpretational point of view. We use simulations to calculate the power of a GPC analysis for a range of treatment effects in a putative comparative experiment, either a matched comparison between a group receiving an experimental treatment and a natural-history cohort, or when feasible, a randomized control trial comparing an experimental treatment with a standard of care [8].

## Methods

### Generalized pairwise comparisons

The method of generalized pairwise comparisons (GPC) to analyze data from comparative clinical trials has been described in detail elsewhere [3]. Essentially, each individual in the intervention group is compared against each individual in the control group, and the resulting pair is assigned a pairwise score of + 1, − 1 or 0 depending on whether the comparison favors treatment (a “win”), control (a “loss”) or neither (“neutral”). The net benefit is estimated by calculating the sum of the pairwise scores for all possible pairs, divided by the number of pairs. The net benefit is the net probability that a random subject has a better outcome in one group than in the other: it is equal to zero if there is no preference for either treatment or control, to + 1 if all patients in the treatment group do better than all patients in the control group, and to − 1 in the opposite situation. One advantage of the net benefit is that its estimation and interpretation is independent of the type of outcome considered, which can therefore be binary (for instance, success/failure), ordered categorical (for instance, none, mild, moderate, severe), continuous (for instance, a score ranging from 0 to 100), time-to-event (for instance, overall survival), etc. GPC can be implemented using a threshold of clinical relevance, whereby the comparison in a pair is considered neutral if the difference between the outcomes of the patient in the treatment group and the patient in the control group is less than this threshold. For multiple outcomes, GPC can be extended using either a prioritized or a non-prioritized analysis. For the non-prioritized analysis, all the outcomes can be weighted equally, in which case the pairwise score is the average of the pairwise scores for all outcomes. For the prioritized analysis, all the outcomes can be classified following a pre-specified ordering of interest, in which outcomes lower in the hierarchy are only assessed in a pair when the pairwise comparison of the previous outcome led to a tie. Therefore, each pairwise score is calculated from the outcome of highest priority that classifies the pair as a win or a loss in a hierarchical fashion that follows the prespecified ordering. Additional file 1: Appendix I provides further details about GPC and the net benefit.

### Natural-history cohort

The data we used for the simulations came from a prospective natural-history cohort of 23 subjects from 5 centers in Brazil, France, Germany, the Netherlands, and the United Kingdom [24]. Subjects included in the cohort had a documented MPS IIIA diagnosis, were 2 to 9 years old (median age 4 years old), had no other serious co-morbidity, and were medically stable without potential disease-modifying medicinal product.

Data were collected at 6 onsite visits at baseline (assessment day 0), and 6, 12, 18 and 24 months (± 14 days) from baseline. The baseline assessment included a neurocognitive and adaptive behavior assessment, Sanfilippo specific behavioral and disability assessment [17] and quality-of-life questionnaires. Changes from baseline were assessed in terms of cognitive function and adaptive behavior using Vineland Adaptive Behavior Scales, Second Edition (VABS-II) for “expressive language”, “Daily living skills (eating)” and “gross motor (walking)”. The Children’s Sleep Habit Questionnaire (CSHQ) was used to evaluate sleep disturbances. The subject’s and parents’ quality-of-life (pain scale) was evaluated using Infant Toddler quality of life questionnaire (ITQoL) and semi-structured interviews of parents.

Bayley Scales of Infant Development, Third Edition (BSID-III) was used to compute the cognitive Development Quotient (DQ) and select 7 patients among the Natural History Cohort with a score > 50% (Table 1).

**Table 1:** Median and range of the baseline score of patients in the natural history cohort

Domains	Natural history scores at baseline (median, range)
Expressive language (VABS-II)	Median 63, range 22–162
Daily living skills (VABS-II)	Median 30.5, range 28–47
Gross-motor (VABS-II)	Median 58, range 0–74
Sleep (CSHQ)	Median 4, range 0–10
Pain (ITQOL)	Median 75, range 25–100

### Simulations

**Fig. 1:**
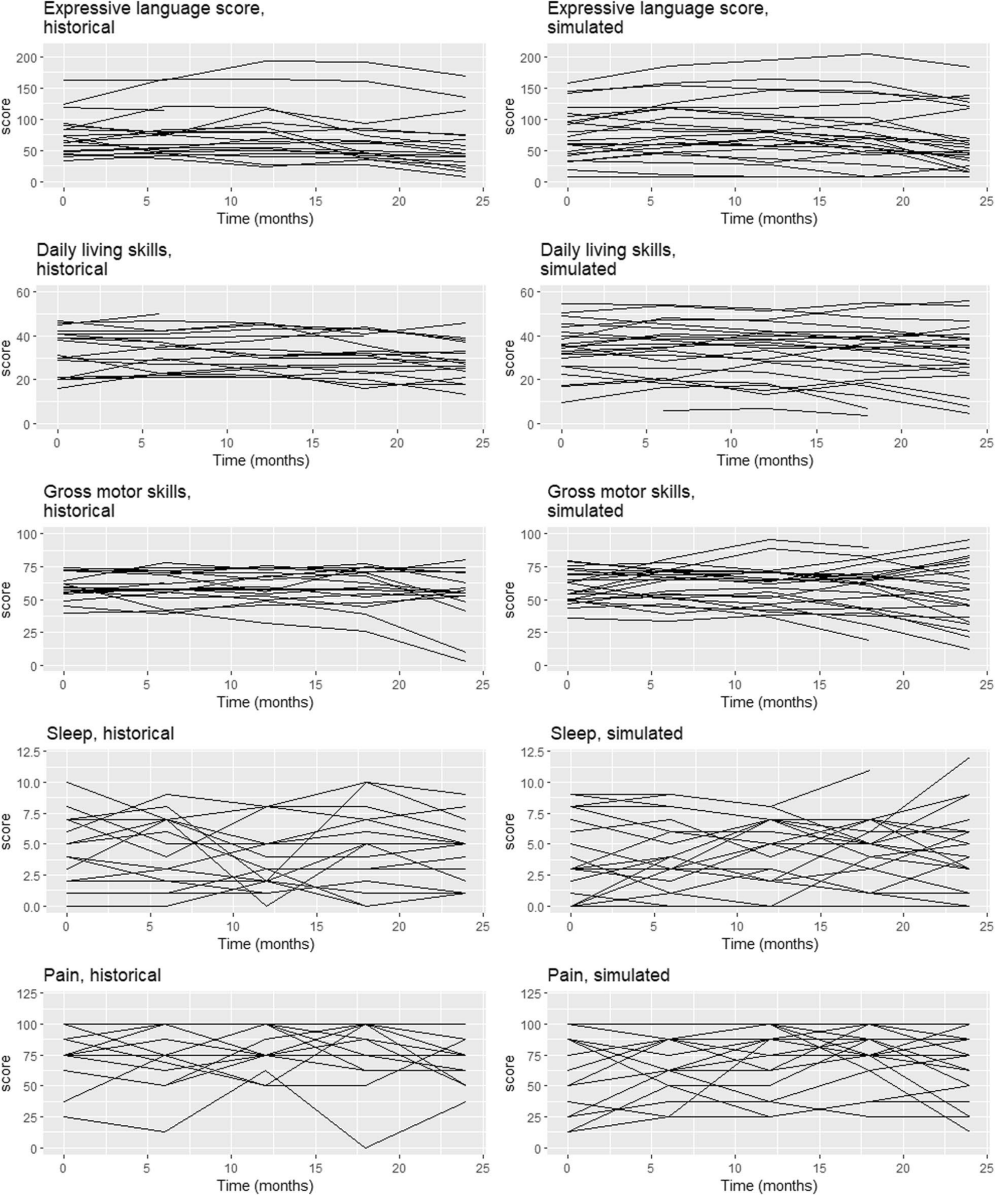
Comparison of the evolutions of individual outcomes over 24 months in the historical data (left column) versus simulated data for the control group (right column). Each line represents one patient

The simulations considered a multi-domain analysis consisting of five outcomes related to the expressive language, daily living skills, and gross-motor, sleep and pain domains. These outcomes were selected because they all represent key domains which parents/caregivers have reported as important symptoms of MPS IIIA to be targeted with future therapies [15]. We used mixed-effects modeling, including both linear and quadratic trends over time, for the data generation in order to fully capture the natural evolution of the disease. The parameters for these models were estimated using the available information from the natural-history cohort. A joint covariance matrix for all five models was also estimated based on the natural-history data. Defining such a joint structure for variability allows the random effects for a same patient to be correlated across the outcomes. Figure 1 shows a comparison between the evolutions of outcomes in historical data *vs.* simulated data for the control group. Visually, the simulated data for the control group adequately mimic the natural-history data.

In our simulations, we assumed a comparison between two groups of 23 subjects (as in the available natural-history cohort). The control group was simulated as explained above (Fig. 1). The parameter of interest for each of the five domains was the rate of change in score over time, defined as the difference in scores from baseline to 2 years, divided by observation time (24 months). This parameter is the slope of a linear trend in score over time. The experimental treatment group was simulated by assuming a shift in the rate of change in score over time. This shift was based on expert opinion about meaningful magnitudes of change (treatment effects) on the various outcomes considered. Table 2 displays the observed rates of change in score over time for each of the five domains in the control group, and the corresponding slopes in the experimental treatment group. Note that the rates of change may depend on the subject’s age, at least for some domains such as expressive language and daily living skills. Given that the limited data we had to conduct simulations, we ignored this dependency. We assumed the same variance–covariance structure for both groups (treated group and natural-history cohort), which could also be subject to criticism.

**Table 2:** Average rate of change in score over time in the control group and in the experimental treatment group. Note that for the outcomes related to the expressive language, daily living skills, gross-motor and pain domains, larger values of the rates of change are considered as better outcome. The inverse is true for the outcome related to the sleep domain

Rate of change (per year) in score over time	Control group	Experimental treatment group
Expressive language (VABS-II)	1.31	3.53
Daily living skills (VABS-II)	0.10	0.42
Gross-motor (VABS-II)	0.28	0.94
Sleep (CSHQ)	− 0.01	− 0.47
Pain (ITQOL)	− 0.21	0.50

We generated 10,000 datasets with a sample size of 46 observations, equally divided into two groups. We didn’t assume any missing information as, given the rarity of the disease, every effort possible is made to follow-up patients carefully. Each of these datasets was analyzed using either non-prioritized (with equal weights across outcomes) or prioritized GPC, with the following list of priorities defined following the expert opinions and feedback from the patients’ families on what matters the most: (1) expressive language, (2) daily living skills, (3) gross motor, (4) sleep and (5) pain (this list of priorities will be referred to henceforth as 12,345). Caregivers feedback also stressed that it is difficult to prioritize these outcomes as the impact and expectations will be different depending on where the child is situated in their disease trajectory.

We compared the two groups based on the rates of change in scores of the five domains over time. Furthermore, we conducted analyses over a range of thresholds of clinical relevance, varying similarly for each outcome, and expressed in terms of the fractions of standard deviations of the distributions of the rates of change for the corresponding outcome.

The p-values for the analyses were based on the permutation distribution [3]. As we simulate assuming the presence of a treatment effect, the power of each test is reflected by the empirical rate of rejection of the null hypothesis of no treatment effect among the 10,000 simulated datasets. We performed sensitivity analyses considering several other possible lists of priorities for the prioritized GPC in order to evaluate possible changes in test results with changing order of priorities.

### Software

Software to implement generalized pairwise comparisons is available in the R [16] package BuyseTest [14], which can be freely downloaded from GitHub and CRAN.

## Results

**Fig. 2:**
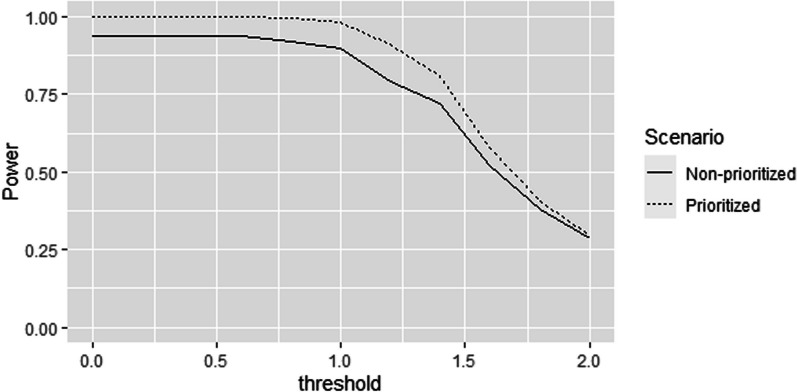
Comparison of the power for prioritized and non-prioritized GPC analyses with threshold values increasing similarly for all outcomes (expressed in standard deviations of the linear trend)

Figure 2 presents the power of the main GPC analysis under either non-prioritized, or prioritized approach, based on the list of priorities (12,345) described above. The non-prioritized approach including all outcomes of interest leads to a lower power across all thresholds of clinical relevance, as compared to a prioritized approach taking expressive language as the outcome of highest priority. The power becomes more similar for high threshold values.

**Fig. 3:**
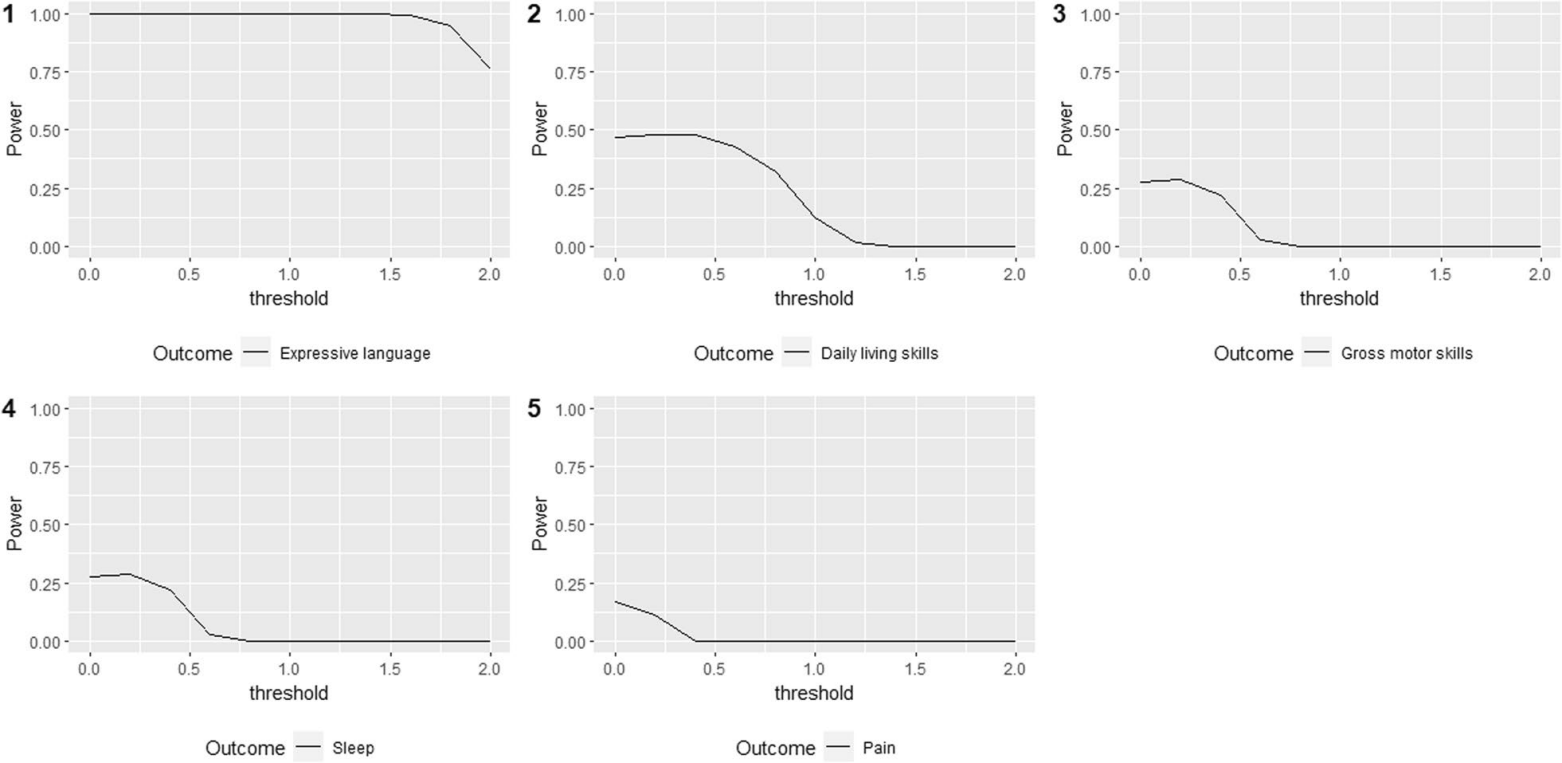
Marginal power of the 5 individual outcomes of interest

Figure 3 shows the marginal power of GPC for each individual outcome. The expressive language, which is the outcome of highest priority in the prioritized analysis (12,345), has very high power when analyzed alone.

**Fig. 4:**
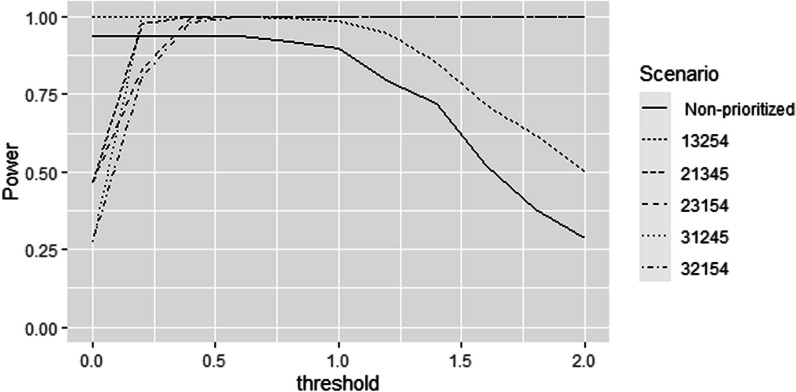
Power of prioritized GPC analyses. The numbers in scenario descriptions correspond to the combination (in decreasing priority from left to right) of the following outcomes: 1-expressive language, 2-daily-living skills, 3-gross-motor function, 4-sleep and 5-pain; with threshold values increasing similarly for all outcomes (expressed in standard deviations of the linear trend)

Figure 4 shows the power of several other possible lists of priorities. In cases where the highest importance is assigned to an outcome with a low marginal power, small threshold values lead to a low power. However, as the threshold increases for all outcomes, there is increasingly less contribution from the low-power high-priority outcome, and more information included from the lower priority outcomes with high marginal power, leading to an increase in the overall power of the prioritized GPC.

## Discussion

We have performed a simulation study in order to investigate the power of two possible multivariate approaches to detection of a treatment effect based on GPC. We have created hypothetical trials in the setting of MPS IIIA, a rare disease which affects multiple aspects of a child’s development, based on data from a natural history study of MPS IIIA. We focused on five main domains: expressive language, daily-living skills, gross-motor function, sleep and pain. All of these aspects play a significant role in the natural history of the disease and impact the quality of life of the patients and their families.

This multi-domain approach is valuable, particularly to the rare disease community where disease phenotypes are complex and vary depending on each patient’s age and disease trajectory, as well as on the time at which treatment is started in this trajectory. Because of the progressive nature of many rare diseases, historically many patients were excluded from clinical trials if their disease had already progressed substantially. This further compounds the difficulty in conducting clinical trials and in having enough statistical power to detect differences in outcomes of interest.

Our simulations show that, in the majority of cases, the prioritized approach leads to a larger overall power as compared with the non-prioritized approach. The non-prioritized approach considers the average treatment effect across all five outcomes, hence the treatment effect of the most powerful outcome is diluted and leads to a lower proportion of rejected null hypotheses of no treatment effect. The opposite is seemingly true only in scenarios where the largest contribution to the overall Net Benefit comes from the outcomes with a very low marginal power. While in our simulations the prioritized approach was generally superior to the non-prioritized GPC, caution should be exercised when generalizing our results: the power of the prioritized GPC will not always be higher than that of the non-prioritized approach. The power of the prioritized GPC can be further influenced not only by marginal treatment effects, but also by correlations between the individual outcomes [7]. Our simulations also showed that the power of the non-prioritized approach becomes more similar to the power of the prioritized approach for high threshold values. This happens because with large thresholds, most of the pairs are classified as “neutral”, and the number of remaining informative pairs (i.e., pairs that are classified as a win or a loss) is insufficient either to allow large differences between the prioritized and non-prioritized settings, or to provide high power for either. Overall, the results of our simulations should be considered illustrative of the methodology, as these depend on the specific assumptions that were made for the treatment effect, resulting from discussions with key-opinion leaders.

The major advantage of prioritized outcomes is that the choice of priorities can potentially be decided by the patient and/or the family, thus enabling a patient-centric analysis to be performed. The flexibility allowed by the prioritization may also be attractive for a disease that evolves over time, such that the outcomes considered most important may also change with the child’s age and degree of disease progression already experienced by the child at the time of initiating therapy. On the other hand, the need to pre-specify a list of priorities for a trial aimed at registration of an experimental drug or treatment may not always be straightforward. Moreover, the selection of domains and their prioritization should involve qualitative research into understanding patients and/or their caregivers needs and expectations from treatment, as a function of the degree of impairment at the time of starting therapy. The non-prioritized approach has its place in situations where each outcome is equally important, and one would like to evaluate the joint effect of treatment on all of them.

Tandon and Kakkis [19] have proposed a multi-domain responder index to capture clinically meaningful changes across multiple domains, which is close in spirit to, but technically different than, the GPC approaches (prioritized and non-prioritized) presented in this paper. The multi-domain responder index uses the treatment-induced change in the score of each individual patient to assign them a “responder” status if the change exceeds a minimally important difference (MID). Numerically, the status is set to + 1 if the change is an improvement exceeding MID, to − 1 if the change is a worsening exceeding MID, and to 0 otherwise. The overall responder status of a patient is the sum of the scores across all outcomes of interest, and as such it has more statistical sensitivity than a single responder analysis [5]. This approach is very attractive for treatment-induced changes in scores, i.e., differences between the score at baseline and the score at a later time suitable to assess treatment effects. The GPC approach is more general as it uses pairwise comparisons for any outcome type (not just changes in scores, for instance the time to an event) between treated patients and control patients, with thresholds of clinical relevance playing a similar role for pairwise comparisons as the minimally important difference for the change in score within a patient.

Table 3 summarizes the advantages and limitations of the various approaches.

**Table 3:** Advantages and limitations of three methods of analysis of multi-domain scores

Method	Main advantage	Main limitation
GPC with non-prioritized outcomes [13]	Can be applied to outcomes of all types	Statistically significant treatment effects may be due to small differences
GPC with prioritized outcomes [3]	Prioritizes outcomes and allows thresholds of clinical relevance to be specified	Net treatment benefit may be difficult to understand (in probabilistic terms)
Multi-domain responder index [19]	Has better statistical power than a single responder analysis	Requires ability to define “responder” status for all outcomes

Our simulations have several important limitations. We have ignored the effect of age on the rates of change of the scores for the five domains considered. Further simulations could be conducted to include an age dependency, but these simulations should ideally be based on a larger set of natural-history data. We have focused our attention on the average rate of change over a 2-year period, which ignores clinically important time dependencies, for instance larger changes occurring earlier in the disease trajectory. Finally, we have expressed thresholds of clinical relevance as quantiles of the distribution of each outcome. Such a distribution-based approach is convenient because it makes all domains comparable in the analysis (which can be especially important for a non-prioritized approach in which all domains are considered with equal weights). However, the approach illustrated in our simulations could just as easily use absolute changes considered to be clinically meaningful in each domain, just as in the development of a multi-domain responder index [19]. Considerations driving the choice between distribution-based thresholds versus anchor-based thresholds fall beyond the scope of the present paper [9]. Once again, qualitative research would be needed to understand expectations of patients and/or their caregivers, but the approach proposed here seems consistent with the need to capture the effects of new interventions on several outcomes in order to maximize clinical relevance as well as statistical power in the design of clinical trials [5, 12].

## Conclusions

Multivariate analyses using GPC are a particularly interesting tool in the rare-disease setting because they allow the analysis of a large set of outcomes, possibly related to various aspects of the disease evolution and patient quality of life. Besides, they are based on raw measurements instead of a single summary measure which inevitably results in a loss of information. While the statistical relevance of including several outcomes may sometimes be questioned, especially in situations where the joint analysis leads to a lower power than focusing on one specific marginal analysis, the clinical relevance of assessing the impact of treatment on several domains is more in line with how patients and their caregivers will make real world decisions about acceptance of a new treatment’s risks and benefits.

### Supplementary Information

**Additional file 1: Appendix.** Generalized pairwise comparisons and the Net Benefit.

## Data Availability

The code that supports the findings of this simulation study is available on https://osf.io/d9f5p/?view_only=00f44207122649f0b0254b8057e10bb9.

## Abbreviations

BSID-III: Bayley Scales of Infant and Toddler Development, Third Edition
CA: Chronological age
DA: Developmental age
DQ: Development quotient
GPC: Generalized pairwise comparisons
ITQoL: Infant and Toddler Quality of Life
MID: Minimally important difference
MPS III: Mucopolysaccharidosis Type III (Sanfilippo syndrome)
MPS IIIA: Mucopolysaccharidosis Type IIIA (Sanfilippo syndrome type A)
VABS-II: Vineland Adaptive Behavior Scales, Second Edition—Expanded Interview
