# Supplementary material for: Generalized pairwise comparisons of prioritized outcomes are a powerful and patient-centric analysis of multi-domain scores
Source: Orphanet J Rare Dis. 2023 Oct 12;18:321. doi: 10.1186/s13023-023-02943-8 (PMC10571482; doi:10.1186/s13023-023-02943-8)
Supplement: Supplementary file 1 — Additional file 1: Appendix. Generalized pairwise comparisons and the Net Benefit. [file 13023_2023_2943_MOESM1_ESM.docx]

**SUPPLEMENTARY MATERIAL**

**Appendix 1**

**Generalized pairwise comparisons and the Net Benefit**

**Pairwise comparisons**

Assume we wish to compare a group of patients receiving some intervention (called “treatment”) with a group of patients receiving standard of care (called “control”). Denoted the outcome of interest $X$ and $Y$in the treatment group and the control group, respectively. Pairwise comparisons are carried out by forming all possible pairs of patients, taking one patient from each group [1]. A pairwise score is defined as follows for the *i*^th^ patient in the treatment group and the *j*^th^ patient in the control group:

$$u_{ij}=\left\{ \begin{matrix} +1 if X_{i}\succ Y_{j} \\ -1 if X_{i}\prec Y_{j} \\ 0 otherwise \end{matrix} \right.$$

where the symbols “ $\succ$” and $"\prec"$ denote superiority and inferiority, respectively.

The concepts of superiority and inferiority depend on the type of variable considered, but they can easily be defined for binary variables, for numerical variables (*e.g.*, a larger value for a continuous outcome). The pairwise score $u_{ij}$ is equal to 1 if the pair favors treatment (“win”), to -1 if the pairs favors control (“loss”), and to 0 if the pair favors neither treatment nor control (“tie”) [4].

**Multiple prioritized outcomes**

Assume that there are two outcomes of interest, which can be prioritized because an improvement in one of these outcomes is clearly preferred to an improvement in the other outcome. The outcomes are again denoted $X$ and $Y$ in the treatment and the control groups, respectively, with priorities denoted by subscripts 1 or 2 (1 being higher priority than 2). The pairwise score is defined as follows for the *i*^th^ patient in the experimental group and the *j*^th^ patient in the control group:

$$u_{ij}=\left\{ \begin{matrix} +1 if X_{1,i}\succ Y_{1,j} or (X_{1,i}≍Y_{1,j}\mathrm{and}X_{2,i}\succ Y_{2,j}) \\ -1 if X_{1,i}\prec Y_{1,j} or (X_{1,i}≍Y_{1,j}\mathrm{and}X_{2,i}\prec Y_{2,j}) \\ 0 otherwise \end{matrix} \right.$$

where the symbols “ $\succ$” and $"\prec"$ denote superiority and inferiority, respectively, and $≍$ denotes neutrality on the scale of the variables. The pairwise score generalizes easily to any number of prioritized outcomes of any type. The advantage of using prioritized outcomes, besides intuitive appeal, is that the correlation between the outcomes is taken into account [2].

**Multiple non-prioritized outcomes**

Assume that there are several (say, “$m$”) outcomes of interest that cannot be prioritized, but that could potentially be weighted [3, 5]. The pairwise score for the *k*^th^ outcome is defined as follows for the *i*^th^ patient in the experimental group and the *j*^th^ patient in the control group:

$$s_{k,ij}=\left\{ \begin{matrix} +1 if X_{k,i}\succ Y_{k,j} \\ -1 if X_{k,i}\prec Y_{k,j} \\ 0 otherwise \end{matrix} \right.$$

and the pairwise score for the *m* outcomes is defined as:

$$u_{ij}=\frac{1}{\sum_{k=1}^{m} w_{k}}\sum_{k=1}^{m} w_{k}s_{k,ij}$$

where “ $w_{k}$” denotes the weight assigned to the *k*^th^ outcome. Weights are often taken equal for all outcomes, *i.e.*, $w_{k}=\frac{1}{m} \forall k$.

**Thresholds of clinical relevance**

For outcomes that are ordered (including continuous outcomes), a threshold of clinical relevance can be specified so that a difference smaller than the threshold is ignored in pairwise comparisons. For example, a difference of less than 2 points on a 10-point scale might be considered too small to be of clinical relevance. In this case the definition of the pairwise score is defined as follows for the *i*^th^ patient in the treatment group and the *j*^th^ patient in the control group:

$$u_{ij}=\left\{ \begin{matrix} +1 if X_{i}-Y_{j}>\tau\\ -1 if X_{i}-Y_{j}< - \tau\\ 0 otherwise \end{matrix} \right.$$

where “ $\tau$” denotes the threshold of clinical relevance.

**The Net Benefit**

The Net Benefit is estimated as the mean pairwise score over all the pairs that can be formed between one patient from the treatment group and one patient from the control group:

$$\hat{\Delta} ={\sum_{i=1}^{n_{T}} \sum_{j=1}^{n_{C}} u_{ij}}/{n_{T}.n_{C}}$$

where $\hat{\Delta}$denotes the estimated Net Benefit, $n_{T}$ denotes the number of patients in the treatment group and $n_{C}$ the number of patients in the control group. For a single outcome, the Net Benefit Δ represents the probability that a random subject in the treatment group does better than a random subject in the control group, minus the probability of the opposite situation. For a single outcome with a threshold of clinical relevance, the Net Benefit Δ represents the probability that a random subject in the treatment group does better by at least the threshold of clinical relevance than a random subject in the control group, minus the probability of the opposite situation. For multiple prioritized outcomes, the Net Benefit Δ represents the probability that a random subject in the treatment group does better than a random subject in the control group, either for the outcome of highest priority, or, in case of a tie for the outcome of highest priority, for the outcome of next highest priority, and so on, minus the probability of the opposite situation. For multiple non-prioritized outcomes, the Net Benefit Δ represents the (weighted) average probability, calculated across all outcomes, that a random subject in the treatment group does better than a random subject in the control group, minus the probability of the opposite situation.

**STATEMENTS AND DECLARATIONS**

**Ethics approval**

The study P3-LYS-SAF that provided the historical data was an observational study. It received approval from the following Ethics Committees:

- Brazil: Comitê de Ética em Pesquisa (CEP) do Hospital de Clínicas de Porto Alegre (HCPA)
- Germany: Ethics Committee of the Hamburg Medical Association
- France : Comité de Protection des Personnes Ile de France 5
- The Netherlands: Academic Medical Centre, University of Amsterdam Medical Research Ethics Committee
- United Kingdom: London - Queen Square Research Ethics Committee, HRA NRES Centre Manchester

**Consent to participate**

Written informed consent was obtained from the parents or legal guardians.

**Consent for publication**

Not applicable.

**Availability of data and materials**

The code that supports the findings of this simulation study is available on <https://osf.io/d9f5p/?view_only=00f44207122649f0b0254b8057e10bb9> .

**Competing Interests**

Vaiva Deltuvaite-Thomas, Samuel Salvaggio and Marc Buyse were employees at IDDI at the time of writing this paper. Vaiva Deltuvaite-Thomas, Mickaël De Backer, Samuel Salvaggio and Marc Buyse are members of the BENEFIT consortium. Samantha Parker and Marie Deneux were employees of Lysogene SA at the time of writing this paper. Lynda E. Polgreen and Cara O’Neill has received consulting fees from Lysogene.

**Funding:**

Vaiva Deltuvaite-Thomas, Mickaël De Backer, Samuel Salvaggio and Marc Buyse are members of the BENEFIT research consortium, which was partially funded by the region of Wallonia (BioWin Consortium Agreement No 7979).

**Author Contributions**

The study conception and design: SS, MB, MDB, VDT. Simulation study programming and analysis were performed by VDT and MDB. QC of the study code: SS. The first draft of the manuscript was written by VDT. SP, MD, LEP, CO’B provided expert input on the MPS IIIA and the endpoints of interest and their hierarchy. All authors commented on previous versions of the manuscript. All authors read and approved the final manuscript.

**Acknowledgements**

The authors gratefully acknowledge comments received from Dr Julie Eisengart, of the Department of Pediatrics, University of Minnesota, Minneapolis, MN.

**REFERENCES**

[1] Buyse M. Generalized pairwise comparisons of prioritized outcomes in the two-sample problem. Stat Med 2010; 29: 3245-57.

[2] Buyse M, Saad ED, Peron J, Chiem JC, De Backer M, Cantagallo E, Ciani O. The net benefit of a treatment should take the correlation between benefits and harms into account. J Clin Epidemiol 2021; doi:<https://doi.org/10.1016/j.jclinepi.2021.03.018>, 2021.

[3] O’Brien PC. Procedures for comparing samples with multiple endpoints. Biometrics 1984; 69:1079–87.

[4] Pocock SJ, Ariti CA, Collier TJ, Wang D. The win ratio: a new approach to the analysis of composite endpoints in clinical trials based on clinical priorities. European Heart Journal 2012; 33: 176-82.

[5] Verbeeck J, Spitzer E, de Vries T, van Es GA, Anderson WN, Van Mieghem NM, Leon MB, Molenberghs G, Tijssen J. Generalized pairwise comparison methods to analyze (non)-hierarchical composite endpoints. Stat Med 38:5641–56, 2019.
